# 

**Published:** 1864-07

**Authors:** 


*Contents.*

ORIGINAL COMMUNICATIONS .......... 97

1. Case of Traumatic Femoral Aneurism, Treated by Digital Compression.—Ligation afterwards of the External Iliac Artery, reported by Surg. Jackson Chambliss. 2. Healing of Gun-Shot Wounds by First Intention, by Surgeon Middleton Michel. 3. Case of Gun-Shot Wound of the Knee-Joint: Ball lodged in the Head of the Tibia; Extracted through the Joint Six Months after the Accident; Recovery with a Useful Limb—by Surgeon R. A. Kinloch. 4. On Vaccination and Variolous Diseases, by Surgeon O. Kratz. 5. Operations in Reparative Surgery, by Surgeon Chas. Bell Gibson.

EDITORIAL .......... 106

Indigenous Remedies of the South.

ORIGINAL ABSTRACTS AND SELECTIONS .......... 108

On the Superiority of Chopart's Operation and Excision of the Ankle in all Cases Admitting of their Performance, by Surgeon Henry Hancock.

INTERESTING ANALYSES OF ARTICLES IN FOREIGN JOURNALS .......... 111

Surgical Experience at St. Bartholomew's, by Surgeon Frederick C. Skey. Perseverance. On Vaginal Lithotomy. Relative Rank of Medical Officers in the British Service.

